# Heterologous Expression of Immature Forms of Human Islet Amyloid Polypeptide in Yeast Triggers Intracellular Aggregation and Cytotoxicity

**DOI:** 10.3389/fmicb.2020.02035

**Published:** 2020-09-03

**Authors:** Ana F. Raimundo, Sofia Ferreira, Maria I. Farrim, Cláudia N. Santos, Regina Menezes

**Affiliations:** ^1^ iBET ‐ Instituto de Biologia Experimental e Tecnológica, Oeiras, Portugal; ^2^ CEDOC ‐ Chronic Diseases Research Center, Faculdade de Ciências Médicas, Universidade Nova de Lisboa, Lisbon, Portugal; ^3^ ITQB-NOVA ‐ Instituto de Tecnologia Química e Biológica António Xavier, Universidade Nova de Lisboa, Oeiras, Portugal

**Keywords:** islet amyloid polypeptide, islet amyloid polypeptide-induced toxicity, oligomerization, protein aggregation, immature islet amyloid polypeptide, yeast model

## Abstract

Diabetes is a major public health issue that has attained alarming levels worldwide. Pancreatic aggregates of human islet amyloid polypeptide (IAPP) represent a major histopathological hallmark of type 2 diabetes. IAPP is expressed in β-cells as pre-pro-IAPP (ppIAPP) that is first processed to pro-IAPP (pIAPP) and finally to its mature form (matIAPP), being released upon glucose stimulation together with insulin. Impairment and overload of the IAPP processing machinery seem to be associated with the accumulation of immature IAPP species and the formation of toxic intracellular oligomers, which have been associated with β-cell dyshomeostasis and apoptosis. Nevertheless, the pathological importance of these immature IAPP forms for the assembly and cytotoxicity of these oligomers is not completely understood. Here, we describe the generation and characterization of unprecedented *Saccharomyces cerevisiae* models recapitulating IAPP intracellular oligomerization. Expression of green fluorescent protein (GFP) fusions of human ppIAPP, pIAPP, and matIAPP proved to be toxic in yeast cells at different extents, with ppIAPP exerting the most deleterious effect on yeast growth and cell viability. Although expression of all IAPP constructs induced the formation of intracellular aggregates in yeast cells, our data point out the accumulation of insoluble oligomeric species enriched in immature ppIAPP as the trigger of the high toxicity mediated by this construct in cells expressing ppIAPP-GFP. In addition, MS/MS analysis indicated that oligomeric species found in the ppIAPP-GFP lysates contain the N-terminal sequence of the propeptide fused to GFP. These models represent powerful tools for future research focused on the relevance of immature forms in IAPP-induced toxicity. Furthermore, they are extremely useful in high-throughput screenings for genetic and chemical modulators of IAPP aggregation.

## Introduction

Diabetes represents a major social and economic burden. The International Diabetes Federation estimated that, globally, 463 million people were living with diabetes in 2019, and this number is predicted to attain 700 million by 2045 (International Diabetes Federation, 2019).

Type 2 diabetes mellitus (T2DM) is a multi-factorial disease with multiple contributing factors for its onset. The main features of the pathology are high glycemia due to inadequate insulin secretion/action and/or β-cell deficiency (Kahn et al., 2014).

About 90% of T2DM individuals exhibit deposits of islet amyloid polypeptide (IAPP), also referred as amylin, in pancreatic islets (Westermark and Grimelius, 1973). IAPP is a hormone co-expressed, -processed, and -secreted with insulin by pancreatic β-cells upon glucose stimulation. Its physiological effects are mainly related to regulating gastric emptying, controlling adiposity and satiation and acting synergistically with insulin in stabilizing post-prandial blood sugar levels (Lutz, 2010; Akter et al., 2016; Zhang et al., 2016). IAPP is synthesized as a 89-residue pre-pro-IAPP (ppIAPP) from which the signal peptide (SP) is cleaved in the endoplasmatic reticulum (ER) to form pro-IAPP (pIAPP). This prohormone is then processed in the late Golgi complex by cleaving two flanking peptides by protein convertases (PCs) 1/3 and 2, giving rise to mature IAPP (matIAPP; Westermark et al., 2011). IAPP is stored in secretory granules of β-cells together with insulin, in a tightly controlled ratio, to ensure its solubility (Kahn et al., 1993; Zhang et al., 2016).

As a consequence of their simultaneous production, the boost in insulin production following hyperglycemia, observed under pathological conditions, is accompanied by hyperamylinemia, which is *per se* a trigger of IAPP oligomerization (Westermark et al., 2011). In addition, some evidences indicate that the overload of the processing machinery prompts the secretion and accumulation of immature IAPP forms (Marzban et al., 2006; Paulsson et al., 2006; Chen et al., 2018). In fact, these intermediate species are claimed as more amyloidogenic than matIAPP (Paulsson and Westermark, 2005), they were found in cells models lacking PC (Marzban et al., 2006) and in intracellular amyloid deposits of transgenic animals and human diabetic individuals (Paulsson et al., 2006). Furthermore, the ratio of circulating pIAPP/IAPP was found to be altered in individuals with T2DM and impaired glucose tolerance (Zheng et al., 2010). Despite this, the pathological relevance of these immature IAPP forms for the assembly of cytotoxic oligomers remains to be elucidated.

As IAPP species accumulate, oligomers start to form inside of the pancreatic β-cells. These oligomers have several known toxic effects, namely, inhibiting cell proliferation, deteriorating β-cells function (Shigihara et al., 2014), increasing ER stress (Huang et al., 2007), and provoking defects in autophagy (Rivera et al., 2014), among others. When the accumulation of these oligomeric species surpasses a certain threshold and cell defense mechanisms are no longer able to resolve them, there is the formation of larger amyloid fibrils, which lately culminates with the buildup of amyloid deposits that are hallmarks of the disease.

Several *Saccharomyces cerevisiae* models have been previously reported as powerful tools to study protein aggregation phenomena in other pathological contexts, namely, neurodegenerative diseases (Outeiro and Lindquist, 2003; Willingham et al., 2003; D’Angelo et al., 2013; Oliveira et al., 2017). In this study, we describe novel yeast models expressing ppIAPP, pIAPP, and matIAPP fused to green fluorescent protein (GFP), which recapitulates intracellular aggregation, leading to marked toxicity and growth impairment, particularly in ppIAPP-GFP expressing cells. We show that increased cytotoxicity of ppIAPP-GFP is associated with the intracellular accumulation of insoluble oligomeric species enriched in immature ppIAPP. Furthermore, we confirmed that oligomeric species found in the ppIAPP-GFP lysates contain at least the N-terminal sequence of IAPP propeptide fused to GFP.

## Materials and Methods

### Strains and Plasmids

BY4741 MATa *his3*Δ*1 leu2*Δ*0 met15*Δ*0 ura3*Δ*0* (obtained from EUROSCARF) was the yeast strain used in this study. The plasmids used are listed in Supplementary Table S1. To construct p426-matIAPP-GFP, p426-Gal-aSyn-GFP (Outeiro and Lindquist, 2003) was digested with *Spe*I/*Hin*dIII to remove aSyn. The complementary DNA (cDNA) sequence of matIAPP was amplified by PCR and cloned into this vector using the In-Fusion Cloning kit (TAKARA Clontech). The same strategy was used to generate the remaining constructs upon replacement of the matIAPP sequence by the cDNA sequences corresponding to ppIAPP SP, ppIAPP, and pIAPP. Yeast transformation procedures were carried out as indicated using lithium acetate standard method (Gietz and Schiestl, 1991).

### Growth Conditions

Synthetic dropout (SD)-glucose medium [0.67% (w/v) yeast nitrogen base (YNB) without amino acids (Difco, United States), 0.77 g/L single amino acid dropout CSM_-URA_ (MP Biomedicals, United States), and 2% (w/v) glucose (Sigma-Aldrich, United States)] was used for growth of cells transformed with p426-derived plasmids. A pre-inoculum was prepared in SD-raffinose medium [0.67% (w/v) YNB, 0.77 g/L CSM_-URA_, 1% (w/v) raffinose (Sigma-Aldrich, United States)], and cultures were incubated overnight at 30°C under orbital shaking. Cultures were diluted in fresh medium and, unless stated otherwise, they were incubated under the same conditions until the optical density at 600 nm (OD_600_) reached 0.5 ± 0.05 (log growth phase). The following equation was used to synchronize the cultures: ODi × *V*i = ODf/[2^(*t*/gt)^] × *V*f, where ODi is the initial optical density of the culture, *V*i is the initial volume of culture, ODf is the final optical density of the culture, *t* is the time (usually 16 h), gt is the generation time of the strain, and *V*f is the final volume of culture. Readings were performed in 96-well plates using a Biotek Power Wave XS plate spectrophotometer. Cell cultures were diluted as indicated for each assay. In all experiments, repression or induction of constructs was carried out in SD-glucose medium and SD-galactose [0.67% (w/v) YNB, 0.77 g/L CSM_-URA_, 2% (w/v) galactose (Sigma®, Germany)], respectively.

### Flow Cytometry

Cell cultures were diluted to OD_600_ 0.1 ± 0.01 in SD-galactose and incubated at 30°C for the indicated time points under orbital agitation. Cells were incubated with propidium iodide (PI) at a final concentration of 5 μg/ml for 30 min at 30°C under orbital agitation and protected from light. Flow cytometry was performed using a CyFlow Cube 6 (Sysmex Partec GmbH, Goerlitz, Germany), equipped with a blue solid-state laser (488 nm), green fluorescence channel (530/30 nm), and orange red fluorescence channel (610/20 nm). Data analysis was performed using FlowJo software. A minimum of 1,00,000 events were collected for each experiment. Cell doublets exclusion was performed based on Forward-A and -W scatter parameters. Results were expressed as the percentage of PI^+^ and GFP^+^ positive cells as compared to the control.

### Growth Assays

For the growth curves, cultures were diluted to OD_600_ 0.05 ± 0.005 in SD-glucose and SD-galactose and incubated at 30°C with shaking for 24 h. Growth was monitored hourly by measuring OD_600_ using a Biotek Power Wave XS Microplate Spectrophotometer (Biotek®, Winooski, United States). A model-free spline (nonparametric) and a model fitting (parametric) approaches were used to calculate the growth parameters in the R software. The package *grofit* (Kahm et al., 2010) was used to adjust a model-free spline, and the parameters, maximum cell growth (μm) and length of the lag phase (lag time), were estimated from the spline fit. The same package was also used to adjust to a model-based curve, and the parameters were estimated from the best fit model. The 95% confidence intervals (95% CIs) were calculated *via* bootstrapping for both model-free spline and model-based fits. The results of the analysis are represented by the best model curve with 95% CIs for each strain compared to the control strain (not expressing IAPP).

### Phenotypic Assays

For the phenotypic growth assays, cells were grown in SD-raffinose medium at OD_600_ = 0.4 ± 0.02, and the OD_600_ was adjusted to 0.1 ± 0.01. Serial dilutions were performed with a ratio of 1:3, and 5 μl of each dilution was spotted onto solid SD-glucose and SD-galactose media. Growth was recorded after 48 h incubation at 30°C. Images were acquired using Chemidoc™ XRS and ImageLab® software.

### Protein Extraction and Immunoblotting

Cell cultures were diluted to OD_600_ = 0.1 ± 0.01 in SD-galactose and incubated at 30°C for the indicated time points under orbital agitation. Cells were collected by centrifugation for 4 min at 2,500 *g*, the pellets were resuspended in Tris-buffered saline solution [TBS; Tris 2.4 g/L (Carl Roth GmbH, Germany), 8 g/L NaCl (PanReac Applichem, Germany), pH 7.6] supplemented with protease and phosphatase inhibitors, cells were disrupted with glass beads (3 cycles of 30 s in the vortex and 5 min on ice), and cells debris were removed by centrifugation at 700 *g* for 3 min. Total protein was quantified using the MicroBCA kit (Thermo Fisher Scientific, United States) according the manufactures’ instructions. Samples were incubated at 95°C for 10 min before sodium dodecyl sulphate-polyacrylamide gel electrophoresis (SDS-PAGE). Ten micrograms of total proteins was loaded and resolved in Mini-Protean TGX Gels (Bio-Rad, United States). Gels were transferred to PVDF membranes using the Trans-Blot® SD semi-dry transfer system (Bio-Rad, United States). Membranes were activated with methanol and blocked for 1 h at room temperature with 5% (w/v) bovine serum albumin (BSA, Sigma-Aldrich, United States) dissolved in TBS-T [Tris 2.4 g/L (Carl Roth GmbH, Germany), 8 g/L NaCl (PanReac Applichem, Germany), and 0.1% (v/v) Tween 20 (Sigma-Aldrich, United States)]. The primary antibodies against GFP (Neuromabs, California, United States), IAPP (Sigma-Aldrich, United States), and Pgk1 (Invitrogen, United States) were probed overnight at 4°C as indicated in Supplementary Table S2. The membranes were then washed 6 × 5 min in TBS-T and incubated with the appropriated secondary antibody for 1 h at room temperature. The membranes were washed 3 × 10 min in TBS-T and incubated with ECL™ Prime Western Blotting System (GE Healthcare, United States). Images were acquired using Chemidoc™ XRS and analyzed using ImageLab® software.

### Fluorescence Microscopy

Cell cultures were diluted to OD_600_ = 0.1 ± 0.01 in SD-galactose, incubated at 30°C for 12 h under orbital agitation, and centrifuged at 3,000 *g* for 3 min. Slides were prepared using 4 μl of cell suspension, and mixed with 4 μl of low melting point agarose for samples to be analyzed by confocal microscopy. GFP fluorescence was visualized using a Carl Zeiss LSM 710 (for confocal microscopy) or Leica Z2 (for fluorescence microscopy). Images were analyzed using Fiji-ImageJ1.51j8, United States. The number of GFP positive (GFP+) cells and cells with aggregates and determination of aggregates area were evaluated by monitoring at least 800 cells for each condition.

### Triton Soluble and Insoluble Fractions

Triton soluble and insoluble fractions were separated as described before (Tenreiro et al., 2014). Briefly, 200 μg of total protein was incubated with 1% Triton X-100 for 30 min on ice. The protein was centrifuged at 15,000 *g* for 60 min at 4°C. The supernatant was considered the soluble protein fraction and collected. The pellet was resuspended in 40 μl of 2% sodium dodecyl sulphate (SDS) Tris-HCl buffer pH 7.4 by pipetting and 10 s of sonication and considered the insoluble fraction. Equal volumes of soluble and insoluble fractions were loaded and resolved by SDS-PAGE as described above.

### Solubility Computational Predictor

The sequence of ppIAPP-GFP, which is equivalent to the other constructs with the exception of the regions that are removed during processing, was inserted into the free CamSol Intrinsic software (Sormanni et al., 2015, 2017) and the solubility score per residue, for the whole IAPP-GFP sequence, was calculated assuming a pH of 7. Scores higher than 1 indicate highly soluble regions, while scores smaller than −1 suggest poorly soluble ones.

### Filter-Trap Assays

Protein lysates were prepared and quantified as indicated above. Increasing concentrations (5, 10, and 50 μg) of total proteins were diluted in TBS 1% (v/v) SDS, loaded onto a 0.22 μm pore nitrocellulose membrane (GE Healthcare, United States), and pre-equilibrated with TBS in a slot blot apparatus. The samples were allowed to pass through the membrane by vacuum, and the slots were washed twice with TBS 1% (v/v) SDS. The membranes were blocked 1 h at room temperature with 5% BSA dissolved in TBS-T and incubated overnight with anti-IAPP primary antibody at 4°C (Supplementary Table S2). The membranes were washed 6 × 5 min in TBS-T and incubated with ECL™ Prime Western Blotting System (GE Healthcare, United States). Images were acquired using Chemidoc™ XRS and ImageLab® software.

### Mass Spectrometry

Protein extracts were obtained as indicated, and 10 μg of total proteins was loaded and resolved in Mini-Protean TGX Gels (Bio-Rad, United States) in duplicates. One gel was processed for immunoblotting as indicated, and the corresponding signals were excised from the duplicate gel previously stained with InstantBlue™ (Sigma-Aldrich, United States).

Mass spectometry sample preparation and analysis was carried out at Clarify Analytical (Portugal). Excised gel fragments were first washed with ultrapure water (100 μl) and incubated twice in 50% acetonitrile (ACN) triethylammonium bicarbonate (TEAB) 50 mM at room temperature, under 1,500 rpm, for 5 min. Following washing with 100% ACN (100 μl), gel fragments were subsequently incubated with DTT 25 mM (100 μl) at 56°C for 20 min, and then with IAA 55 mM (100 μl) at room temperature in the dark for 20 min. After washing with ultrapure water (100 μl), gel fragments were incubated twice with 50% ACN TEAB 50 mM (170 μl) at room temperature, under 1,500 rpm, for 5 min. Following washing with 100% ACN, the digestion was initiated upon trypsin (20 μl of enzyme in 0.01% surfactant in TEAB 50 mM) addition. About 0.01% surfactant in TEAB 50 mM (30 μl) was added, whereupon the mixture was incubated at 37°C for 3 h and then at 50°C for 1 h. Samples were centrifuged at maximum speed for 10 s, and the supernatant were subsequently transferred to new microcentrifuge tubes to which 50% ACN 1% TFA (20 μl) was added. Extracts were combined with the digestion, and gel was discarded. Following centrifugation at maximum speed for 10 min, samples were concentrated and resuspended in 0.1% TFA (10 μl) for ZipTip™ cleanup/enrichment (according to standard protocol) before mass spectrometry (MS) analysis. Protein identification was performed by nanoLC-MS/MS, composed by an Ultimate 3000 liquid chromatography system coupled to a Q-Exactive Hybrid Quadrupole-Orbitrap Mass Spectrometer (Thermo Scientific, Bremen, Germany). Samples were loaded onto a trapping cartridge (Acclaim PepMap C18 100 Å, 5 mm × 300 μm i.d., 160454, Thermo Scientific) in a mobile phase of 2% ACN, and 0.1% formic acid (FA) at 10 μl/min. After 3 min loading, the trap column was switched in-line to a 50 cm by 75 μm inner diameter EASY-Spray column (ES803, PepMap RSLC, C18, 2 μm, Thermo Scientific, Bremen, Germany) at 250 nL/min. Separation was generated by mixing A: 0.1% FA and B: 80% ACN, with the following gradient: 2 min (2.5–10% B), 50 min (10–35% B), 8 min (35–99% B), and 10 min (hold 99% B). Subsequently, the column was equilibrated with 2.5% B for 17 min. Data acquisition was controlled by Xcalibur 4.0 and Tune 2.9 software (Thermo Scientific, Bremen, Germany). The mass spectrometer was operated in data-dependent (dd) positive acquisition mode alternating between a full scan (m*/z* 380-1580) and subsequent HCD MS/MS of the 10 most intense peaks from full scan (normalized collision energy of 27%). ESI spray voltage was 1.9 kV. Global settings: use lock masses best (m*/z* 445.12003), lock mass injection Full MS, and chrom. peak width (FWHM) 15 s. Full scan settings: 70 k resolution (m*/z* 200), AGC target 3e6, maximum injection time 120 ms. dd settings: minimum AGC target 8e3, intensity threshold 7.3e4, charge exclusion: unassigned, 1, 8, >8, peptide match preferred, exclude isotopes on, dynamic exclusion 45 s. MS2 settings: microscans 1, resolution 35 k (m*/z* 200), automatic gain control (AGC) target 2e5, maximum injection time 110 ms, isolation window 2.0 m*/z*, isolation offset 0.0 m/*z*, spectrum data type profile. The raw data were processed using Proteome Discoverer 2.4.0.305 software (Thermo Scientific) and searched against the UniProt database for the *Homo sapiens* Proteome 2019_09 and *S. cerevisiae* Proteome 2019_11. The fasta file for GFP was also included. The Sequest HT and MaxQuant search engines were used to identify tryptic peptides. The ion mass tolerance was 10 ppm for precursor ions and 0.02 Da for fragment ions. Maximum allowed missing cleavage sites was set 2. Cysteine carbamidomethylation was defined as constant modification. Methionine oxidation, protein N-terminus acetylation, methionine loss, and methionine loss plus acetylation were defined as variable modifications. Peptide confidence was set to high. The processing node target decoy PSM validator was enabled with maximum delta (Cn = 0.05) and decoy database search target FDR 1%. Protein label free quantitation was performed with the Minora feature detector node at the processing step. Precursor ions quantification was performed at the processing step, including unique and razor peptides. The precursor abundance was based on intensity.

### Size Exclusion chromatography

Total protein lysates were obtained as indicated and centrifuged at 10,000 *g* for 10 min to remove insoluble particles. Three micrograms of total protein was diluted in 2 ml of TBS buffer and loaded on a Superose 6 10/300 GL column (GE Healthcare, Uppsala, Sweden) using an AKTA system (GE Healthcare, Uppsala, Sweden). The samples were eluted with 1 mM potassium phosphate, 155 mM sodium chloride, 2.9 mM sodium phosphate buffer, pH 7.4 at a flow rate of 0.5 ml/min, and the UV absorbance was monitored at 280 nm. To estimate the molecular weight of the protein samples, Gel Filtration HMW Calibration Kit (GE Healthcare, Uppsala, Sweden) plus Ribonuclease (PanReac AppliChem, Spain) were used. Fractions of 500 μl were collected, precipitated overnight at 4°C in TCA, washed three times in acetone, resuspended in a protein sample buffer [0.12 M Tris-HCl, pH 6.8, 9% (v/v) β-Mercaptoethanol, 20% (v/v) glycerol, 4% (w/v) SDS, and 0.05% (w/v) Bromophenol Blue], and resolved by SDS-PAGE using Mini-Protean TGX Gels (Bio-Rad, United States). Immunoblottings were performed as indicated.

### Statistical Analysis

Statistical analysis was carried out using Graphpad Prism 6 software. Data are mean ± SD of at least three independent biological replicates. Two-way ANOVA with the Dunnett’s multiple comparison test was performed to access differences between the conditions. The *t*-test was used to access differences between the SP-GFP and ppIAPP-GFP conditions in the flow cytometry analysis. Two-way ANOVA with the Tukey’s multiple comparison test was performed to access differences in aggregates size between the conditions.

## Results and Discussion

### Expression of Immature IAPP Forms in Yeast Mediates Higher Toxicity Than Mature IAPP

**Figure 1:**
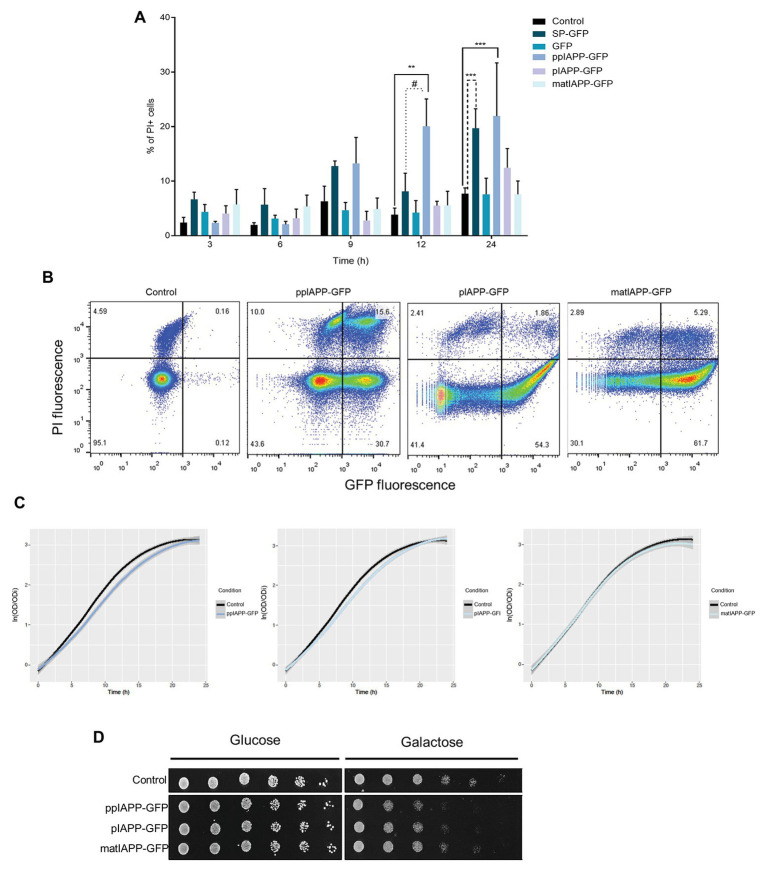
Expression of pre-pro-islet amyloid polypeptide-green fluorescent protein (ppIAPP-GFP) in yeast impairs cell viability and growth. **(A)** BY4741 cells expressing IAPP-GFP fusions and the respective controls were induced with galactose for the indicated time points, and the frequency of propidium iodide (PI) positive cells was assessed by flow cytometry. **(B)** IAPP-GFP vs. PI fluorescence clouds after 12 h galactose induction as determined by flow cytometry. **(C)** Parametric adjustment of growth curves, with confidence intervals (CIs), based on culture optical density at 600 nm (OD_600_) of yeast cells expressing IAPP-GFP fusions, compared to cells that are not expressing the human protein. **(D)** Phenotypic assays of the indicated strains. Cell suspensions were adjusted to the same OD_600_ serially diluted and spotted onto the surface of solid medium containing either glucose or galactose. Representative images are shown, and the values represent mean ± SD from at least three independent experiments. Statistical differences are denoted as ^**^p < 0.01, and ^***^p < 0.001 vs. the control condition; ^#^*p* < 0.05 vs. signal peptide-GFP (SP-GFP).

Some evidences suggest that impairment of ppIAPP processing and the consequent accumulation of intermediate immature forms may contribute to the formation of toxic intracellular oligomers (Paulsson and Westermark, 2005; Marzban et al., 2006; Paulsson et al., 2006). To compare the intracellular effects of IAPP forms in yeast, we generated the chimeric constructs p426-ppIAPP-GFP (ppIAPP-GFP), p426-pIAPP-GFP (pIAPP), and p426-matIAPP-GFP (matIAPP; Supplementary Table S1), in which the respective cDNAs were cloned in frame with GFP under the control of the *GAL1*-inducible promoter. The presence of GFP expressing cells was first confirmed by flow cytometry (Supplementary Figure S1). The considerable standard deviation observed is possibly associated with the expression of IAPP constructs being driven by a multicopy plasmid, which may generate some variability in IAPP levels among biological replicates. Membrane integrity was then determined using PI staining as a measure to infer cell viability. None of the constructs p426 (control), p426-GFP (GFP), and p426-SP-GFP (SP-GFP) affected cell viability up to 12 h induction of protein expression with galactose (Figure 1A). The ppIAPP-GFP construct mediated high levels of toxicity after 12 and 24 h of induction with galactose. The control construct SP-GFP was shown to be toxic at 24 h (Figure 1A). As at 12 h ppIAPP-GFP-mediated cytotoxicity was shown to be significantly higher than SP-GFP, with minor differences in the number of GFP^+^ cells for all GFP-expressing strains (Figure 1B; Supplementary Figure S1), we selected this time point for further analysis. Nevertheless, 20.1 ± 5.0% of the yeast cells expressing ppIAPP-GFP was PI^+^, compared to only 5.5 ± 0.8% or 5.6 ± 2.6% of cells expressing pIAPP-GFP or matIAPP-GFP, respectively (Figures 1A,B).

The effects of ppIAPP-GFP, pIAPP-GFP, and matIAPP-GFP expression on cellular growth were assessed by means of growth curve analysis (Figure 1C). Cultures of cells expressing the three IAPP forms exhibited a short lag phase (corresponding to about one generation time), compatible with the switch from raffinose to galactose as the sole carbon source in the medium (Supplementary Figure S2A, left panel). Growth remained similar during the first 5 h when the strains expressing ppIAPP-GFP started to grow slightly slower than the control cells. In agreement with the reduction of viability observed in the flow cytometry assays (Figure 1A) after 9 h galactose induction, the growth defects of cells expressing ppIAPP-GFP were evident, whereas for pIAPP-GFP-expressing cells this effect could only be slightly detected at 12 h. In contrast, cells expressing matIAPP exhibited no growth defects (Figure 1C). The growth differences among strains are driven by the reduced “maximum growth rate” of ppIAPP-GFP and pIAPP-strains as compared to the control (Supplementary Figure S2A, right panel).

The deleterious effects of ppIAPP-GFP expression and pIAPP at a lesser extent were further corroborated by phenotypic assays, which clearly showed a dramatic reduction as compared with the control strain (Figure 1D). Although pIAPP-GFP and matIAPP-GFP did not compromise cell viability significantly, as indicated before by the flow cytometry analysis (Figure 1A), these constructs interfere with cellular growth, more pronouncedly in pIAPP-GFP than in matIAPP-GFP. Altogether, these results demonstrate that expressions of the three IAPP forms (ppIAPP, pIAPP, and matIAPP) are toxic to yeast cells at different degrees. Noteworthy, the immature forms, particularly the full-length ppIAPP, are the most toxic.

### Intracellular Aggregates Are Formed in Cells Expressing IAPP

**Figure 2:**
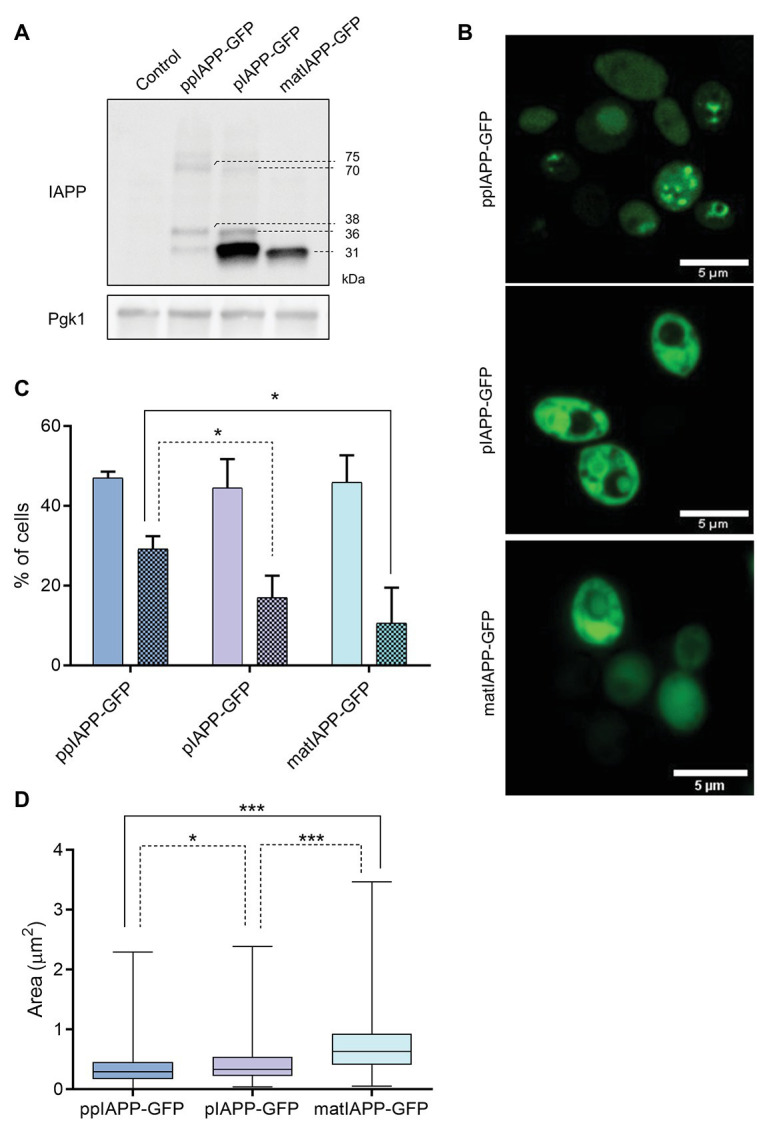
IAPP-GFP fusions expressed in yeast are processed and lead to the formation of intracellular aggregates with different morphologies. **(A)** BY4741 cells expressing IAPP-GFP fusions and the control construct were induced with galactose for 12 h, and proteins were assessed by immunoblotting using anti-IAPP antibody. Pgk1 was used as loading control. **(B)** Confocal fluorescence imaging of ppIAPP-GFP, pro-IAPP (pIAPP)-GFP, and mature IAPP (matIAPP)-GFP cells induced with galactose for 12 h. Scale bars correspond to 5 μm. **(C)** Cells under the same conditions were analyzed by fluorescence microscopy, and the number of fluorescent cells as well as the number of cells containing aggregates was monitored. Full colors: GFP + cells; colored patterns: cells with aggregates. **(D)** Aggregate area was measured in cells containing aggregates. Representative images are shown, and the values represent mean ± SD from at least three independent experiments. Statistical differences are denoted as ^*^p < 0.05 and ^***^p < 0.001.

**Figure 3:**
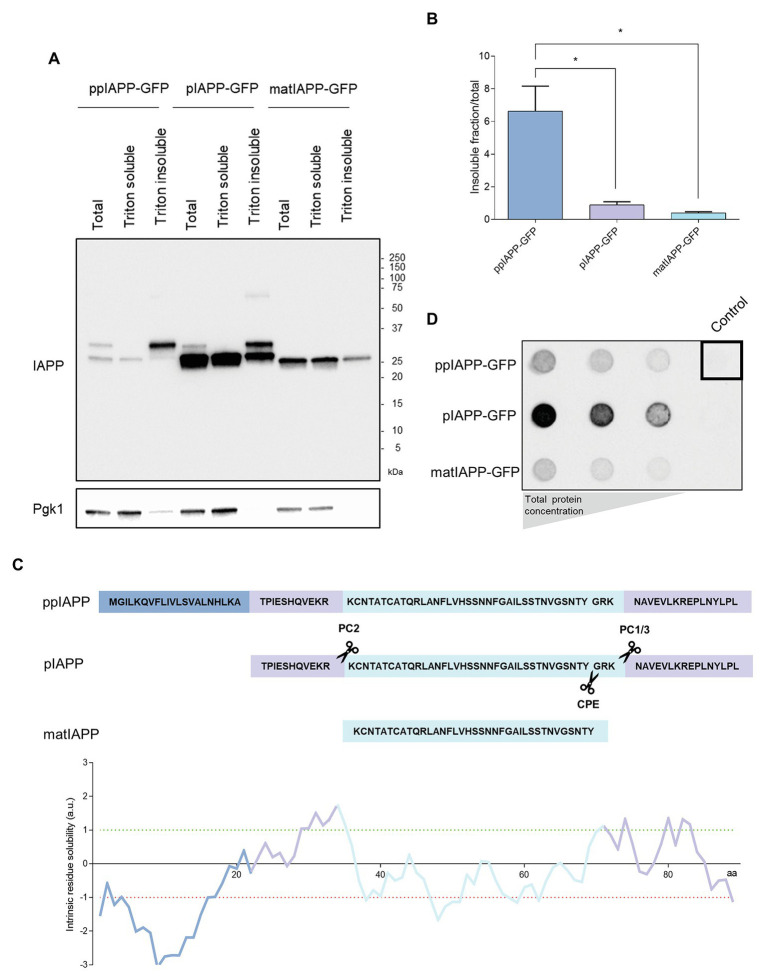
Immature IAPP entities are restricted to Triton insoluble fractions and are differentially retained in filter-trap assays. **(A)** The protein extracts were separated according to their solubility and assessed by immunoblotting using anti-IAPP antibody. Pgk1 was used as loading control. **(B)** The ratio between the insoluble fraction and total protein was calculated for each strain. **(C)** Schematic representation of IAPP processing and solubility. The sequences of the signal peptide (SP; dark blue), the N-terminal and C-terminal flanking regions of the propeptide (medium blue), and the matIAPP (light blue) as well as the processing sites of the prohormone convertases PC2 and PC1/3 are shown. The intrinsic solubility of each amino acid of the full length ppIAPP was predicted computationally (lower panel). **(D)** Filter-trap assays of total protein lysates obtained from ppIAPP-GFP, pIAPP-GFP, and matIAPP-GFP cells induced with galactose for 12 h, and probed with anti-IAPP antibody. Protein concentration loaded in the membranes: 50, 10, and 5 μg (from left panel to right panel). Representative images are shown, and the values represent mean ± SD from at least three independent experiments. Statistical differences are denoted as ^*^p < 0.05.

To understand the mechanisms by which the expression of IAPP immature forms induced higher toxicity in yeast than matIAPP, we next carried out immunoblotting assays. As depicted in Figure 2A, the levels of ppIAPP-GFP, pIAPP-GFP, and matIAPP-GFP proteins were shown to be different among strains, with no significant impact in the number of GFP^+^ cells, as revealed by the flow cytometry analysis (Supplementary Figure S1). Total protein lysates from cells expressing ppIAPP-GFP revealed the presence of a 38 kDa signal compatible with the molecular weight of the full-length ppIAPP-GFP fusion. Remarkably, two additional IAPP-specific signals of ~32 and ~75 kDa were detected in this lysate (Figure 2A). In a similar manner, pIAPP-GFP-protein lysates disclosed the presence of a 36 kDa signal, matching the molecular weight of the pIAPP-GFP fusion and two extra signals of ~32 and ~70 kDa. At this point, we speculated that these signals may represent processing intermediates (~32 kDa signals) and dimers of ppIAPP-GFP and pIAPP-GFP (~70 and 75 kDa signals, respectively). Total protein lysates from cells expressing matIAPP-GFP revealed the presence of a single signal of ~31 kDa and well-matched with the molecular weight of the mature construct. Similar results were observed when the membranes were incubated with anti-GFP antibody (Supplementary Figure S3); however, an immunoreactive signal of ~60 kDa was detected in the lysates of cells expressing matIAPP-GFP. Also, a signal of ~30 kDa, that was not detected by the anti-IAPP antibody, was revealed in the lysates of cells expressing ppIAPP-GFP and pIAPP-GFP. Considering that ppIAPP and pIAPP may be processed by endogenous yeast convertases such as Kex2 (Fuller et al., 1989a,b; Kjeldsen, 2000), this signal may correspond to GFP fused to the C-terminal flanking peptide located downstream of the PC1/3 cleavage site both in ppIAPP and pIAPP (Figure 3C).

As the immunoblotting assays suggested the presence of oligomers in cells expressing ppIAPP-GFP, pIAPP-GFP, and possibly also matIAPP-GFP, we then performed confocal microscopy to follow the subcellular dynamics of chimeric proteins. Cells expressing ppIAPP-GFP displayed a heterogenous distribution of well-defined intracellular aggregates in most of the fluorescent cells (Figure 2B). pIAPP-GFP and matIAPP-GFP cells presented a more diffuse fluorescence pattern throughout the cell and decorated with less sharply defined aggregates. These differences in fluorescence patterns, together with the impairment of cell viability and growth of ppIAPP-GFP strain (Figure 1), may explain the decreased number of GFP^+^ cells after 9 h induction of ppIAPP-GFP expression (Supplementary Figure S1).

Noteworthy, both in pIAPP-GFP and matIAPP-GFP, it is possible to detect aggregates in a non-fluorescent compartment, presumably the vacuole, indicating the accumulation of intravacuolar aggregates.

The subcellular dynamics of aggregates formed in the different strains is possibly associated with the incorporation of the three IAPP forms into different molecular pathways and may explain why the full-length ppIAPP-GFP triggers higher toxicity to yeast cells than the other forms.

We next used fluorescence microscopy to assess the frequency of cells displaying IAPP aggregates (Figure 2C; Supplementary Figure S4). For that, we first counted the number of GFP^+^ cells, which were shown to be statistically similar, in support of the results of the flow cytometry analysis (Supplementary Figure S1). From those, we determined the number of cells bearing aggregates and measured the area of those aggregates. The data confirmed the presence of aggregates in cells expressing all constructs. ppIAPP-GFP cells were the ones displaying the higher number of cells with aggregates as compared to pIAPP-GFP and matIAPP-GFP, which presented similar numbers of cells with aggregates. Furthermore, monitoring of aggregates area indicated that ppIAPP-GFP-expressing cells accumulated aggregates of smaller average area than the other strains (Figure 2D). Furthermore, the frequency analysis distribution of the size of the aggregates indicates that ppIAPP-GFP and pIAPP-GFP have a distribution centered on smaller aggregates, in contrast to matIAPP-GFP which presents a broader distribution, with a bigger percentage of aggregates of larger area (Supplementary Figure S5).

To verify if the fluorescent structures seen in the microscopy analysis represented IAPP aggregates with different properties, the cell lysates were separated in Triton soluble and insoluble fractions. All strains displayed insoluble IAPP aggregates (Figure 3A; Supplementary Figure S6). The quantification of the ratio insoluble/total proteins clearly showed that ppIAPP-GFP strain has a higher proportion of insoluble aggregates compared to the other strains (Figure 3B). Remarkably, the signal corresponding to immature forms of ppIAPP-GFP and pIAPP-GFP could only be detected in the total extracts and, at a higher extent, in the insoluble fraction (Figure 3A; Supplementary Figure S6). This suggests that the aggregates formed by IAPP immature forms are more insoluble than the ones formed by matIAPP. This assumption was corroborated by the computational predictor that applies the CamSol Intrinsic algorithm on the different fusion proteins, defining a solubility score for each one based on the contribution of each amino acid in the protein sequence (Supplementary Figure S7). The predictions indicate that ppIAPP-GFP is the most insoluble of the three forms, with pIAPP-GFP and matIAPP-GFP presenting a similar score. Furthermore, evaluating the intrinsic solubility of each amino acid of the full length ppIAPP individually (which also contains the pIAPP and IAPP), as detailed in Figure 3C, it was possible to assess the high contribution of the SP to ppIAPP insolubility.

To further corroborate these results, total protein extracts were subjected to filter-trap assays, an extensively used tool to assess the formation of intracellular aggregates (Juenemann et al., 2015; Nasir et al., 2015; Cox and Ecroyd, 2017). Surprisingly, lysates from pIAPP-GFP-expressing cells displayed a strong signal revealing the presence of high amounts of IAPP SDS-insoluble aggregates, as opposed to the lysates of cells expressing ppIAPP-GFP and matIAPP-GFP (Figure 3D). However, these results should be interpreted with caution as the protein levels of IAPP-GFP fusions are extremely variable among strains (Figure 2A).

### Identification of Peptides Present in the Putative Oligomeric and Processed IAPP Forms

In addition to the signals clearly corresponding to monomeric forms of ppIAPP-GFP and pIAPP-GFP, the respective lysates also revealed the presence of other IAPP-immunoreactive signals of high and low molecular weight (Figure 2A). To assess the identity of these protein species by nanoLC-MS/MS, the corresponding SDS-PAGE gel fragments were excised (Supplementary Figure S8), and the proteins were digested with trypsin and purified before loading of the peptide mixture on the trapping cartridge. Searching the raw data against the UniProt database for *Homo sapiens*, *S. cerevisiae*, and the fasta file for GFP led to the identification of hundreds of proteins, including unique tryptic peptides for IAPP (Table 1), particularly the peptide [R].NAVEVLKR.[E], corresponding to the C-terminal sequence of IAPP propeptide (Figure 3C), that was detected in the three samples. The internal IAPP peptide [R].LANFLVHSSNNFGAILSSTNVGSNTYGKR.[N] was also identified in sample #3, corresponding to the low molecular weight bands in pIAPP-GFP lysates (Supplementary Figure S8). Analysis using the MaxQuant also identified the peptide [R].LANFLVHSSNNFGAILSSTNVGSNTYGK.[R] in the same sample. As expected, tryptic peptides for GFP were also detected in all samples. These data confirm that the high molecular weight species observed in the ppIAPP-GFP lysates contains the C-terminal end of IAPP propeptide fused to GFP, as it is also immunoreactive for GFP (Supplementary Figure S3). In addition, they suggest that the low molecular weight species observed in the ppIAPP-GFP and pIAPP lysates may represent processing intermediates cleaved by endogenous yeast convertases.

**Table 1:** Identification of IAPP peptides by NanoLC-MS/MS.

Sample No.	Protein	MW (kDa)^a^	PI^b^	*q*-value^c^	Score^d^	Coverage (%)^e^	Peptide^f^	Peptide sequence
#1 HMW ppIAPP-GFP	Human IAPP	9.8	9.8	0.012	2.06	9	1	[R].NAVEVLKR.[E]
#2 LMW ppIAPP-GFP				0.002	3.56	9	2	[R].NAVEVLK.[R]
								[R].NAVEVLKR.[E]
#3 LMW pIAPP-GFP				0	13.66	42	3	[R].NAVEVLK.[R]
								[R].NAVEVLKR.[E]
								[R].LANFLVHSSNNFGAILSSTNVGSNTYGKR.[N]

HMW, high molecular weight signal; LMW, low molecular weight signal.

a Relative molecular mass.

b Isoelectric point.

c Expect value – probability of the observed match between the experimental data, and mass values calculated from a candidate peptide or protein sequence, occur due to random event.

d Mascot score that is calculated by −10Log10(*P*), where *P* is the absolute probability that observed match is random event.

e Percentage of sequences hit for registered full sequence.

f Number of peptide fragments matching the candidate protein.

### IAPP Oligomeric Species of Different Sizes Accumulate in Cells Expressing ppIAPP-GFP

**Figure 4:**
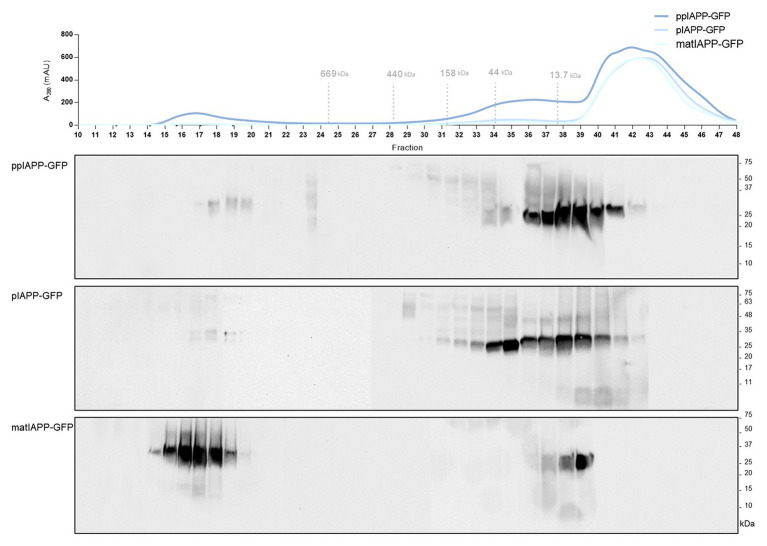
Yeast expressing ppIAPP-GFP accumulates high levels of intracellular oligomers with low molecular weight as compared to pIAPP-GFP and matIAP-GFP strains. The aggregated species formed by the different IAPP-GFP fusion were resolved using size exclusion-chromatography (SEC) after 12 h of cell induction of galactose. Fractions were collected and separated on SDS-PAGE followed by immunoblot analyses. A representative result is shown from at least three independent experiments.

ppIAPP-GFP expressing cells exhibited more cells with aggregates, which seem to have a different nature of the ones accumulated in pIAPP-GFP and IAPP-GFP cells, and that cause a strong impairment of viability and growth. In an attempt to understand the toxic nature of ppIAPP-GFP, we performed size exclusion chromatography, a widely established methodology for the biochemical characterization of protein species. After fractionation of lysates from ppIAPP-GFP, pIAPP-GFP, and matIAPP-GFP expressing cells, we detected IAPP species in different fractions depending on the strain (Figure 4). ppIAPP-GFP and pIAPP-GFP proteins were mostly eluted between fractions 28 and 42, corresponding to low molecular weight species, presumably low-order oligomers and monomers. Stronger signals were detected in fractions 32–35 of pIAPP-GFP extracts, suggesting the accumulation of higher molecular weight species in this strain as compared to ppIAPP-GFP. In both strains, a small amount of even higher molecular weight species (>669 kDa) was also observed in fractions 17–20. Noteworthy, for the matIAPP-GFP strain, most of the protein signals were detected in fractions 14–20. In summary, according to the molecular weight of IAPP species detected, the strains can be ranked as follows: IAPP-GFP > pIAPP-GFP > ppIAPP-GFP. These may comprise the aggregated species detected in previous assays (Figure 2).

Altogether, these results convincingly corroborate the idea that accumulation of insoluble oligomeric species of smaller sizes, enriched in immature ppIAPP, may explain the high toxicity triggered by this construct in cells expressing ppIAPP-GFP.

The toxic nature of IAPP aggregates has been described in several studies linking IAPP to T2DM. Aggregates of synthetic human IAPP are able to induce apoptosis of β-cells from adult pancreas of rats and humans (Lorenzo et al., 1994), and inhibition of IAPP synthesis/aggregation can ameliorate β-cell death in culture human islets (Potter et al., 2009). Aggregation of synthetic IAPP *in vitro* occurs in a concentration-dependent manner (Bailey et al., 2011), suggesting that the levels of IAPP present *in vivo* might be a key contributing factor for aggregates formation. In fact, it has been reported that overexpression of human IAPP in cells can lead to intracellular IAPP aggregation and amyloid formation. Although logical, this process is not so simple, and heterogeneity between β-cells of transgenic models for the formation of detectable human IAPP aggregates was also reported and attributed to differences in chaperone ability, protein trafficking, and capacity to clear misfolded and aggregated proteins (Lin et al., 2007).

The size, nature, and composition of these aggregated species have been a focus of research for a long time. Nonfibrillar IAPP toxic oligomers were detected in two transgenic human IAPP mouse models at the time of maximal loss of β-cell mass. These oligomeric species were found to be confined to β-cells in the perinuclear region and in frequent small discrete deposits, therefore supporting their ability to act on intracellular molecular pathways (Lin et al., 2007). Besides the size of aggregates, findings suggest that deficiencies in processing of immature IAPP forms could be associated with an increase in amyloidogenicity and, consequently, in cytotoxicity. Expression of human pIAPP in GH3 cells lacking prohormone convertases lead to an accumulation of unprocessed and partially processed forms of pIAPP that markedly increased cell death (Marzban et al., 2004). In INS-1 cells, however, expression of human pIAPP did not promote cytotoxicity unless pIAPP expression was high enough to cause an impairment in the processing pathway (Marzban et al., 2006).

In our study, even though all forms of IAPP form aggregates when overexpressed in yeast, those of immature forms cause a more marked toxicity, particularly ppIAPP-GFP. These data bring forward the importance of IAPP processing on the progress of IAPP fibrillization and aggregation, making clear the need for further research.

Furthermore, our data suggest that pIAPP-GFP expression leads to increased formation of aggregates bigger than 0.22 μm but that these are less toxic than the aggregates formed in cells expressing ppIAPP-GFP. This comes in line with findings indicating that smaller oligomers are the drivers of toxicity and not mature fibrils (Janson et al., 1999; Kayed et al., 2007; Haataja et al., 2008; Gurlo et al., 2010; Abedini et al., 2016). Finally, analysis of the solubility of the formed aggregates, as well as *in silico* predictions, indicates that the inclusions have different physicochemical properties. This suggests that aggregates formed by mature or immature forms, or putatively a mixture of species, can contribute differently for the oligomerization process and its consequent toxicity.

Recently, a yeast model expressing a genetically-encoded matIAPP oligomer fused to the SP of yeast Kar2, an ER chaperone required for secretory polypeptide translocation, was described (Kayatekin et al., 2018). In this model, core IAPP-driven pathological mechanisms were uncovered, and powerful insights into the IAPP cytotoxicity were disclosed. As it relies on the expression of a genetically encoded hexamer of matIAPP fused to a yeast SP, important aspects concerning ppIAPP processing, and the contribution of immature IAPP forms to the oligomerization process and cytotoxicity cannot be addressed. In that view, this study describes unprecedented yeasts models expressing the full-length human ppIAPP, the intermediated form pIAPP, and matIAPP fused to GFP, which represent a novel and powerful tool to investigate the molecular mechanisms underlying IAPP proteotoxicity.

## Conclusion

Diabetes represents a major social and economic burden and alarming projections for diabetes incidence emphasize the need of novel therapeutic/detection strategies to minimize disturbing future scenarios. The first high-impact study describing IAPP as an important factor for T2DM was published in 1994. Since then, and although considerable progress has been made, islet amyloid and the precise pathways by which IAPP aggregation causes β-cell death remain somewhat of an enigma in the T2DM field. Opposing to previous ideas, IAPP intracellular oligomers are an early molecular event preceding amyloid deposition and irreversible loss of β-cell functionality. An additional question of some debate is whether IAPP aggregation initiates intracellular or extracellularly. The amyloidogenic properties of human IAPP allied with its overproduction and secretion from β-cells are likely a relevant contributor, but they do not seem to be sufficient for islet amyloid formation. In this perspective, defective trafficking and processing of immature forms of IAPP associated with β-cell dysfunction have been proposed to be a critical trigger for IAPP aggregation. Despite that, the pathological relevance of these unprocessed IAPP species for the assembly of cytotoxic oligomers remains obscure, whereby further studies should be addressed.

The relevance of IAPP proteotoxicity for diabetes pathophysiology and the potential reversibility of oligomers formation make IAPP an attractive target for the design of novel prophylactic, therapeutic, and diagnostic strategies. Our study gives an important contribution to the recent views that point out immature forms of IAPP as the most deleterious for cell survival, highlighting the toxic role of ppIAPP hitherto overlooked. Indeed, the unprecedented models here described represent powerful tools to further investigate the impact of immature forms on IAPP aggregation and proteotoxicity. Unveiling the IAPP aggregation pathway and identifying the toxic species of IAPP is of great importance to the development of new therapies capable of interfering with IAPP toxic oligomerization and consequent amyloid formation. In that regard, the versatility of the models here presented can also be exploited in high-throughput screenings for genetic and chemical modulators of IAPP aggregation.

## Data Availability Statement

The raw data supporting the conclusions of this article will be made available by the authors, without undue reservation, to any qualified researcher.

## Author Contributions

AR, CS, and RM conceived and designed the experiments. AR, SF, and MF performed the experiments. AR, SF, and RM analyzed the data and wrote the paper. All authors contributed to the article and approved the submitted version.

### Conflict of Interest

The authors declare that the research was conducted in the absence of any commercial or financial relationships that could be construed as a potential conflict of interest.
