# Supplementary material for: Heterologous Expression of Immature Forms of Human Islet Amyloid Polypeptide in Yeast Triggers Intracellular Aggregation and Cytotoxicity
Source: Front Microbiol. 2020 Sep 3;11:2035. doi: 10.3389/fmicb.2020.02035 (PMC7496629; doi:10.3389/fmicb.2020.02035)
Supplement: Supplementary file 1 [file Data_Sheet_1.pdf]

Table S1

Table S1: Plasmids used in this study

Plasmid	Description	Source or Reference
p426-Gal-aSyn-GFP	$GAL1_{promoter-}$aSyn-GFP, 2µ, URA	(Outeiro and Lindquist, 2003)
p426*	$GAL1_{promoter}$, 2µ, URA	ATCC® 87341™ **
p426-GFP	$GAL1_{promoter-}$GFP, 2µ, URA	This study
p426-SP-GFP	$GAL1_{promoter-}$SP-GFP, 2µ, URA	This study
p426-ppIAPP-GFP	$GAL1_{promoter-}$ppIAPP-GFP, 2µ, URA	This study
p426-pIAPP-GFP	$GAL1_{promoter-}$pIAPP-GFP, 2µ, URA	This study
p426-matIAPP-GFP	$GAL1_{promoter-}$matIAPP-GFP, 2µ, URA	This study

* Empty plasmid used as controls, ** American Type Culture Collection

Table S2

Table S2: Antibodies used in this study

Antibody	Source	Identifier	Dilution
Rabbit anti-IAPP polyclonal	Sigma-Aldrich (USA)	Cat# HPA053194	1:2500
Mouse anti-GFP	NeuroMabs (California, USA)	Cat# 75-131	1:5000
Mouse anti-Pgk1 monoclonal	Invitrogen (USA)	Cat# 459250	1:1000
Goat anti-mouse polyclonal peroxidase-conjugated	Sigma-Aldrich (USA)	Cat# A5278	1:10000
Stabilized goat anti-rabbit HRP-conjugated	Pierce (USA)	Cat# 1858415	1:5000

Figure S1

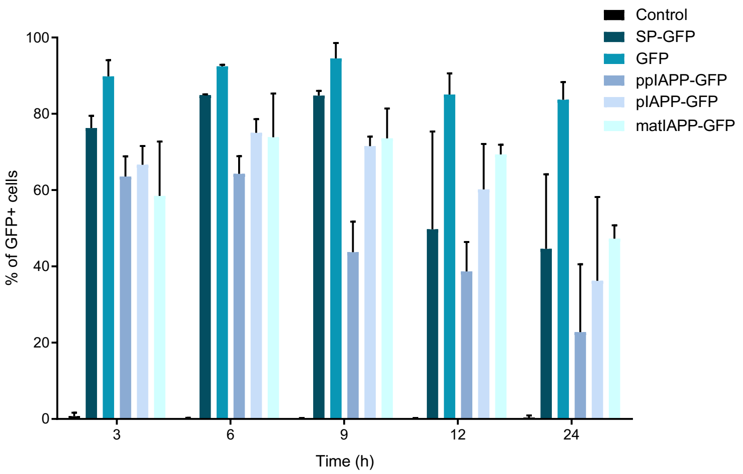

Figure S1: (A) BY4741 cells expressing IAPP-GFP fusions and the respective controls were induced with galactose for the indicated time points and the frequency of GFP cells was assessed by flow cytometry.

Figure S2

A

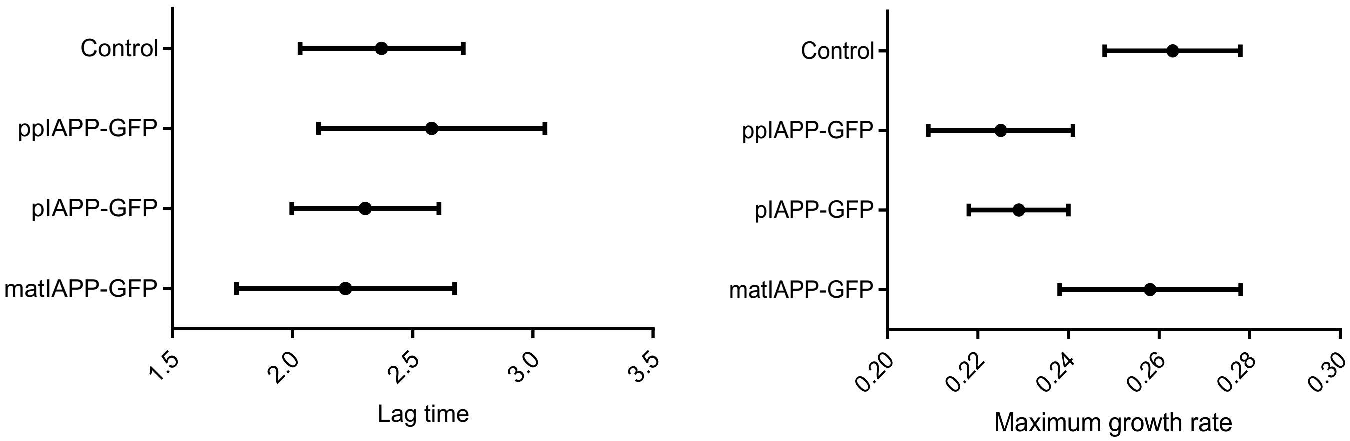

B

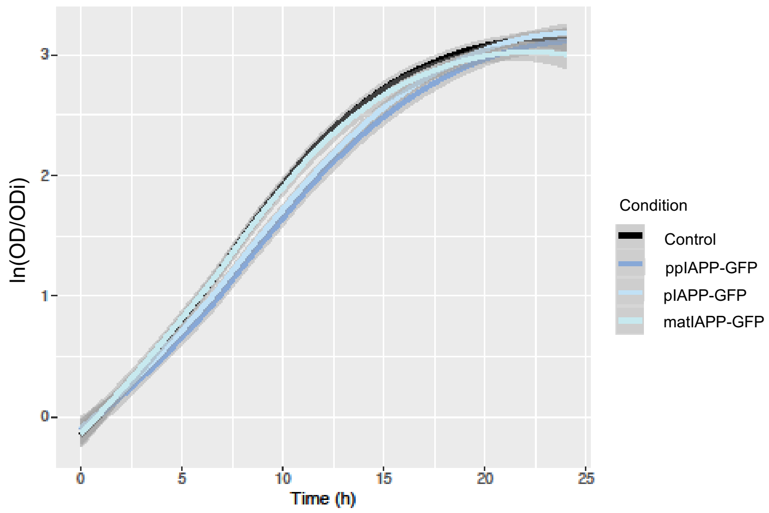

Figure S2: (A) BY4741 cells expressing IAPP-GFP fusions and the control construct were induced with galactose and OD_{600} was monitored hourly. The indicated growth parameters were calculated using the R software. (**B)** Representation of the growth curves of all the strains.

Figure S3

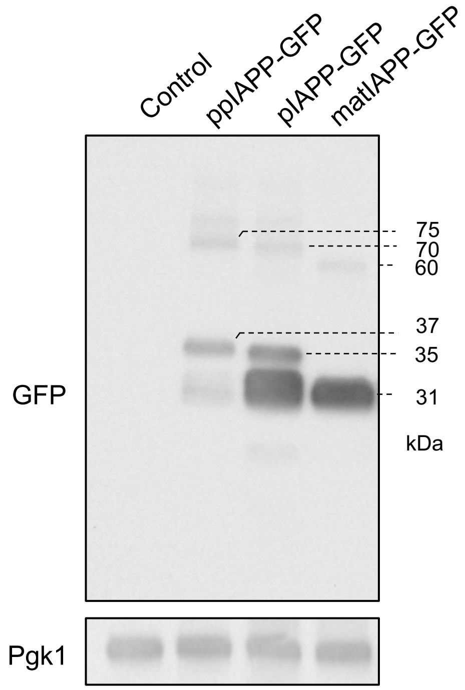

Figure S3: BY4741 cells expressing IAPP-GFP fusions and the control construct were induced with galactose for 12 h and proteins were assessed by immunoblotting using anti-GFP antibody. Pgk1 was used as loading control.

Figure S4

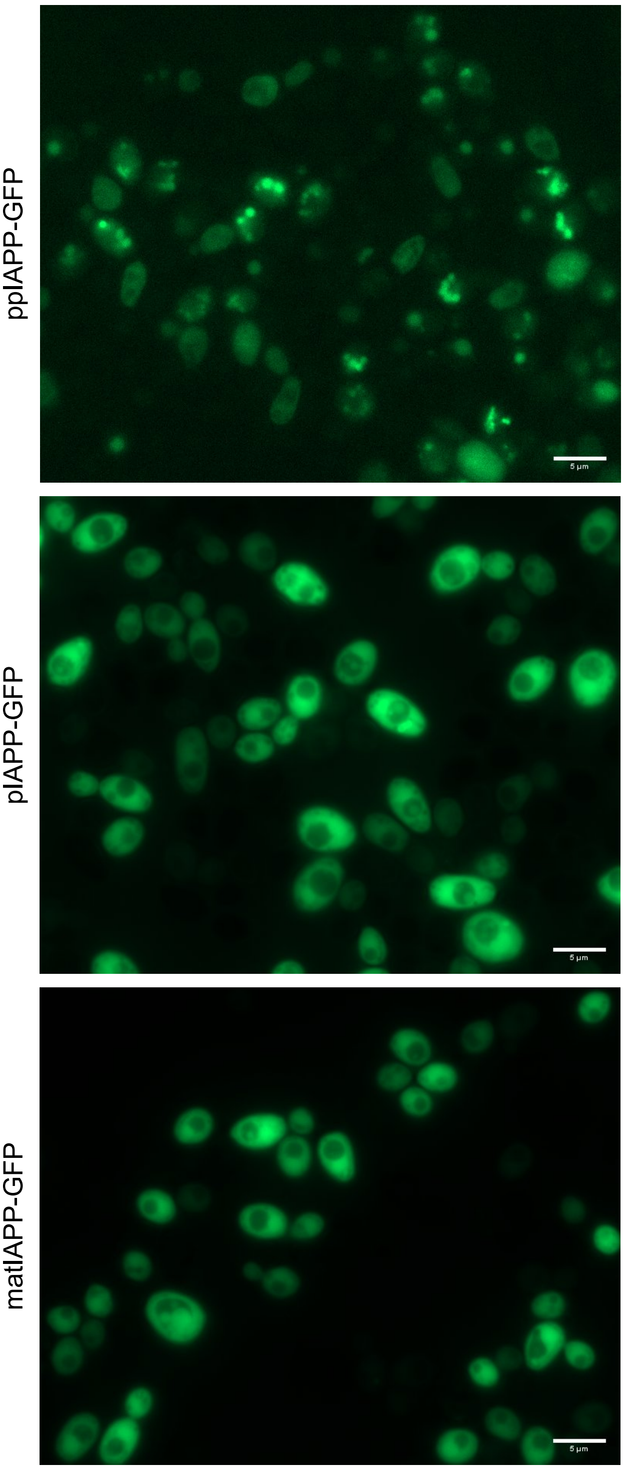

Figure S4: BY4741 cells expressing IAPP-GFP fusions were induced with galactose for 12 h and the number of fluorescent cells as well as the number of cells containing aggregates was monitored by fluorescence microscopy. Scale bars correspond to 5 µm.

Figure S5

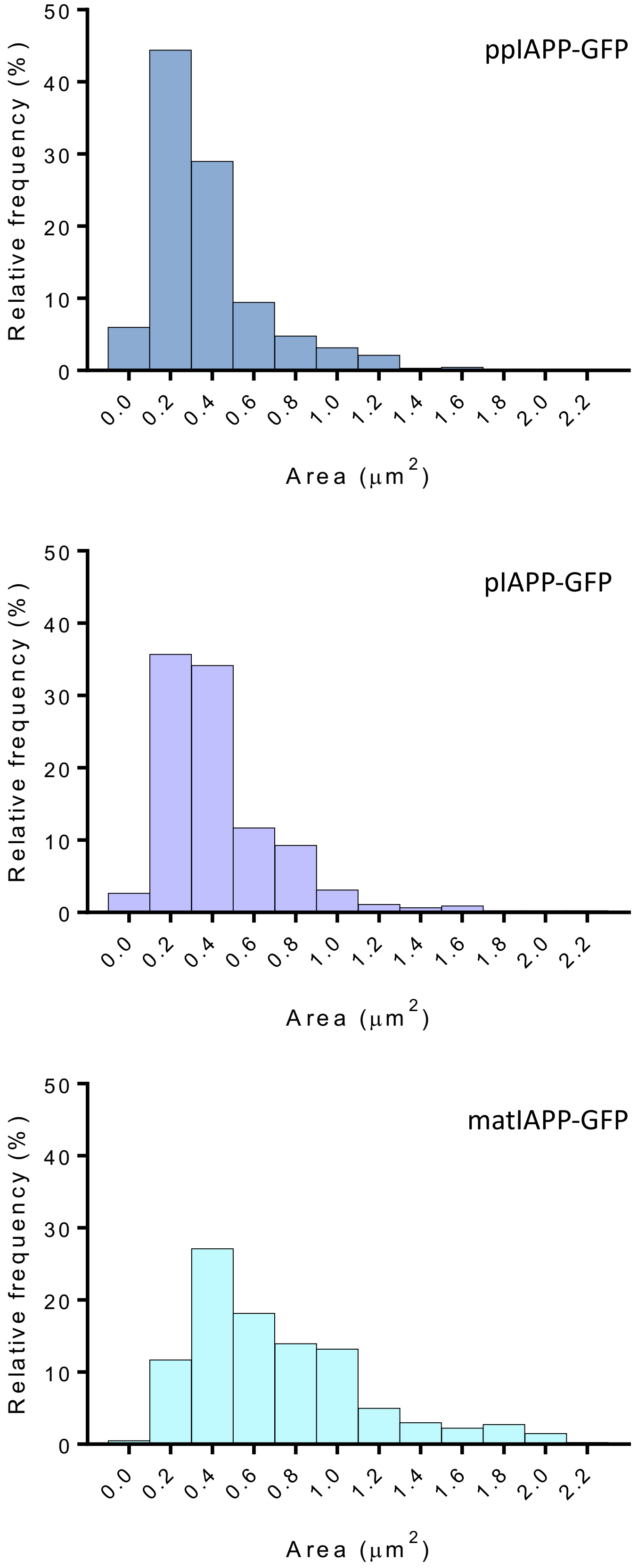

Figure S5: Frequency distribution of the aggregates areas for each of the strains. BY4741 cells expressing matIAPP-GFP fusions were induced with galactose for 12 h and the aggregates area was measured using ImageJ.

Figure S6

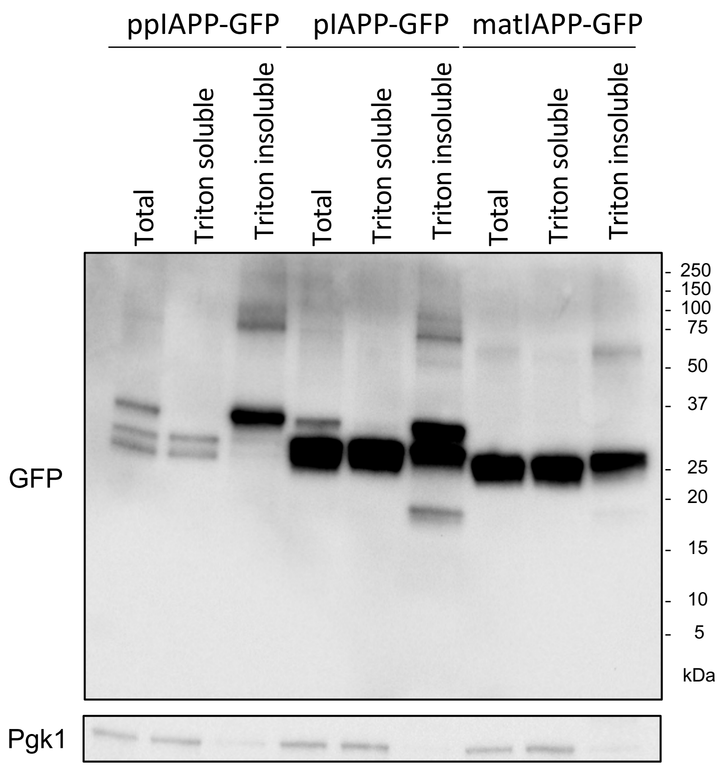

Figure S6: BY4741 cells expressing IAPP-GFP fusions were induced with galactose for 12 h and proteins separated according to their solubility in Triton. Signals were obtained by immunoblotting using anti-GFP antibody. Pgk1 was used as loading control.

Figure S7

A

Protein	Intrinsic solubility score based on the sequence
ppIAPP-GFP	0.037126
pIAPP-GFP	0.672321
matIAPP-GFP	0.589208

B

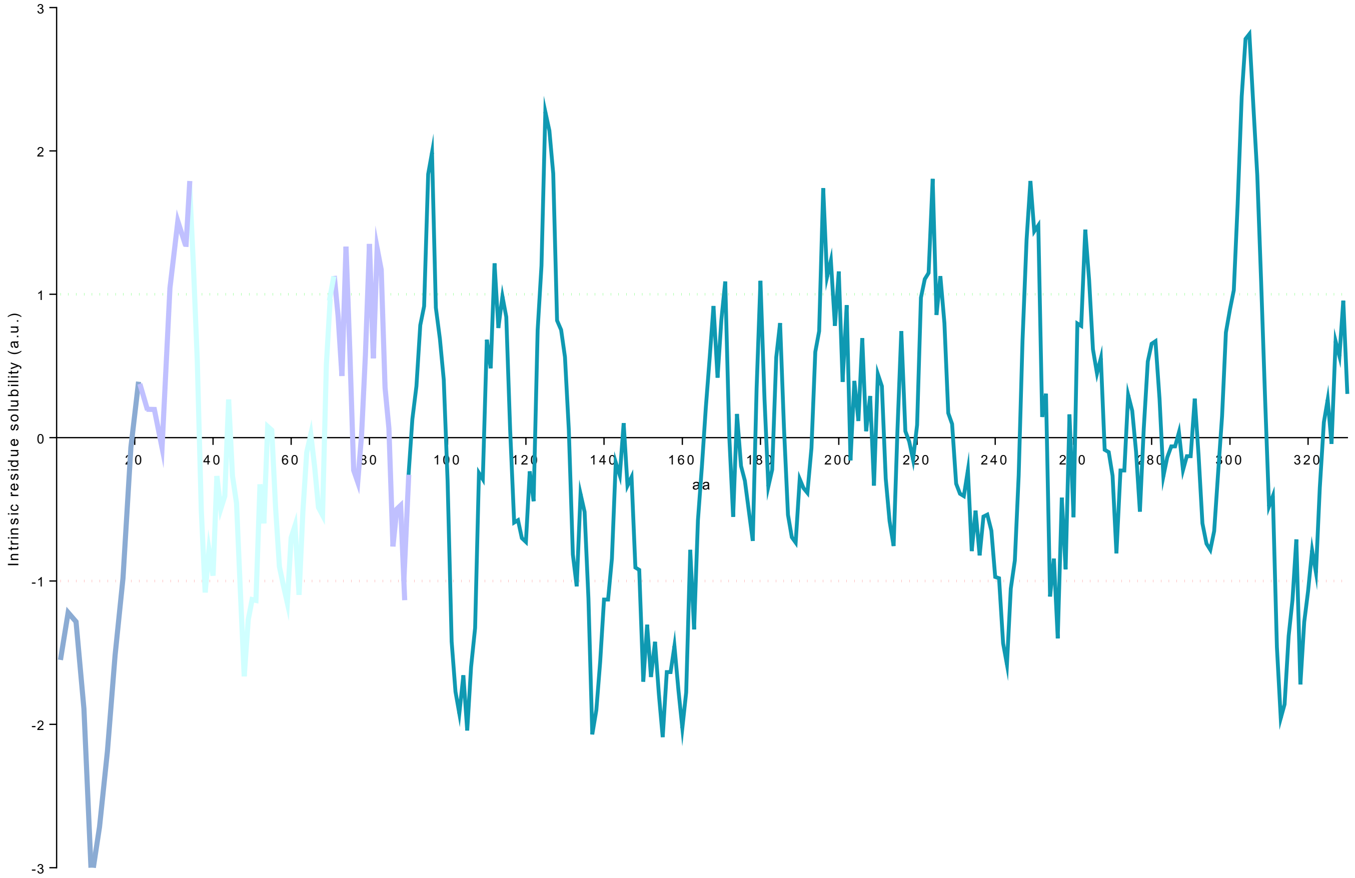

Figure S7: Computational prediction of the solubility of the differently processed IAPP forms by the *CamSol Intrinsic* algorithm. **(A)** Intrinsic solubility scores calculated based on the contribution of each amino acid solubility in the protein sequence. **(B)** Scores higher than 1 indicate highly soluble regions, while scores smaller than -1 suggest poorly soluble ones.

Figure S8

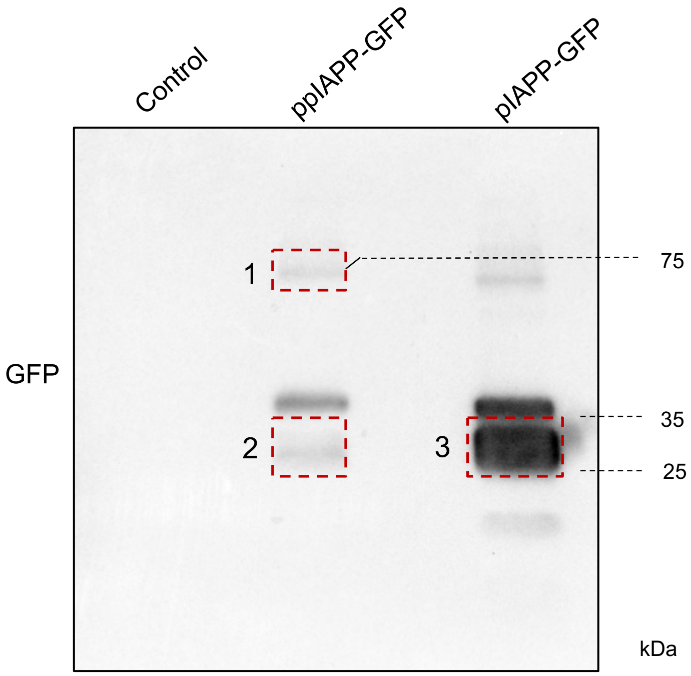

Figure S8: BY4741 cells expressing the indicated IAPP-GFP fusions and the control construct were induced with galactose for 12 h and total proteins resolved in Mini-Protean TGX Gels in duplicates. One gel was processed for immunoblotting (using anti-GFP antibody) and the corresponding signals were excised from the duplicate gel previously stained with InstantBlue^{TM}.
